# Efficacy of traditional Chinese medicine nursing in stroke: a systematic review and meta-analysis

**DOI:** 10.3389/fneur.2025.1657802

**Published:** 2025-08-12

**Authors:** Yanling Chen, Hongyan Li, Zhe Wu, Mingyuan Jiao

**Affiliations:** ^1^Nursing Department, The Second Affiliated Hospital Zhejiang University School of Medicine, Hangzhou, China; ^2^Rehabilitation Department, The Second Affiliated Hospital Zhejiang University School of Medicine, Hangzhou, China; ^3^Research and Teaching Department, Jinhua Maternal and Child Health Care Hospital, Jinhua, China

**Keywords:** stroke, traditional Chinese medicine, nursing, motor function, activities of daily living, meta-analysis

## Abstract

**Objective:**

Some clinical studies have suggested that traditional Chinese medicine (TCM) nursing techniques may promote better recovery in stroke patients compared to conventional neurological nursing. However, high-quality evidence-based research is still lacking. This study selected two outcome indicators—motor function and activities of daily living (ADL)—to systematically evaluate the efficacy of TCM nursing techniques in the rehabilitation of stroke patients.

**Methods:**

Seven major Chinese and English databases were systematically searched. The Fugl-Meyer Assessment (FMA) and the Modified Ashworth Scale (MAS) were used to evaluate motor function, while the Barthel Index (BI) was used to assess ADL. Subgroup analyses were conducted based on study characteristics to explore potential sources of heterogeneity.

**Results:**

A total of 18 studies involving 1,419 patients were included. Meta-analysis results showed that, compared with conventional care, the TCM nursing group demonstrated significantly better outcomes in FMA (SMD = 1.92, *p* = 0.0066), MAS (SMD = −0.82, *p* = 0.0416), and BI (SMD = 1.34, *p* < 0.0001). Meta-regression and subgroup analyses indicated that nursing model, stroke type, and risk of bias were not sources of heterogeneity.

**Conclusion:**

Compared to conventional care, TCM nursing techniques can significantly improve spasticity in stroke patients, and also provide some benefits for limb motor function and ADL. Given the high heterogeneity and poor GRADE assessment of FMA and BI, the above results should be interpreted with caution.

**Systematic review registration:**

https://www.crd.york.ac.uk/prospero/, identifier CRD420251006204.

## 1 Introduction

Stroke is one of the leading causes of disability and death worldwide. According to the WHO, stroke is the second leading cause of death globally and a major contributor to long-term disability. Each year, approximately 12.2 million new cases of stroke occur worldwide, with ischemic stroke accounting for around 62.4% and hemorrhagic stroke for about 37.6% (1). In recent years, the incidence and mortality rates of stroke have shown an upward trend globally, imposing a heavy economic and caregiving burden on both families and society. Moreover, even among stroke survivors, many are left with varying degrees of neurological impairments, such as motor dysfunction, speech difficulties, and cognitive decline, which severely affect their quality of life. Nursing, as an essential part of clinical treatment, plays a crucial role in stroke rehabilitation.

The *American Heart Association*’s guidelines on stroke patient care suggest that conventional neurological nursing can be summarized into four main areas: basic care, early mobilization, psychological and nutritional support. Basic care includes preventing pulmonary and urinary tract infections (aspiration prevention), gastrointestinal and bladder care (constipation, urinary incontinence, and urinary retention), preventing pulmonary embolism and deep vein thrombosis, fall prevention, and skin care (pressure ulcer prevention), with common methods such as repositioning, maintaining patency of tubes, and limb immobilization. Early mobilization aims to prevent joint contractures and maintain joint range of motion. Psychological and nutritional support help to maintain immune function, improve adherence to rehabilitation, and enhance therapeutic outcomes (2, 3). Although these nursing interventions play an important role in the rehabilitation process of stroke patients, they primarily focus on preventing stroke complications, with limited direct effects on improving neurological impairments. Furthermore, conventional neurological nursing tends to follow a relatively uniform model, with limited individualized interventions tailored to the patient’s specific needs. This often leads to slow recovery and, in some cases, increases the risk of stroke recurrence. Therefore, it is imperative to explore more integrated and efficient nursing approaches, making it a key focus in the field of stroke care research.

In recent years, traditional Chinese medicine (TCM) nursing techniques have been widely applied in the rehabilitation of stroke patients and have demonstrated certain clinical advantages. In 2015, the *National Administration of Traditional Chinese Medicine* officially recognized 18 standardized TCM nursing techniques, including acupoint injection, acupoint massage, acupoint application, herbal hot compress, cupping, scraping, herbal iontophoresis, topical herbal application, herbal cold compress, herbal warm-moist compress, herbal enema, herbal fumigation, auricular acupoint pressing, wax therapy, indirect moxibustion, wheat-grain moxibustion, suspended moxibustion, and herbal soaking (4). These methods are grounded in the holistic theory and meridian doctrine of TCM, aiming to regulate the flow of Qi and blood, dredge meridians, and enhance visceral function to promote neurological recovery. Research has shown that acupoint massage can relieve muscle spasticity and improve motor function in paretic limbs (5). Meanwhile, moxibustion and herbal fumigation, through thermal stimulation and transdermal absorption of herbal components, can improve local blood circulation and facilitate neural repair (6, 7).

Compared to conventional nursing, TCM nursing techniques offer several notable advantages. First, TCM nursing techniques are based on the TCM syndrome differentiation and treatment system, which complements individualized care content and aligns with current research trends. Second, some TCM nursing methods, such as massage and moxibustion, not only focus on preventing complications but also have certain neuroprotective effects, which help to promote the recovery of motor function and activities of daily living. Third, TCM nursing techniques are easy to perform, suitable for both inpatient and home care, and highly compatible with rehabilitation training, aligning with current research trends in continuous care.

Although some clinical studies have suggested that TCM nursing techniques may improve neurological function and quality of life in stroke patients, high-quality evidence from clinical trials is still lacking. On one hand, the sample sizes in existing studies are generally small, and there are certain limitations in study design, which may affect the reliability of the findings. On the other hand, some studies have failed to strictly control for confounding factors, and lack randomized controlled trials (RCTs) or large multi-center studies, limiting the generalizability of their conclusions. Against this background, this study aims to provide a comprehensive assessment of the efficacy of TCM nursing techniques in stroke rehabilitation through a systematic review and meta-analysis. The focus will be on their impact on motor function and activities of daily living (ADL), as well as exploring potential factors influencing efficacy. The findings of this study will provide higher-quality evidence for stroke nursing care and offer valuable insights for future clinical practice and research.

## 2 Materials and methods

This study adheres to the Preferred Reporting Items for Systematic Review and Meta-Analysis (PRISMA) 2020 guidelines (Supplementary material 1). PROSPERO registration number: CRD420251006204. The PICOS (Population, Intervention, Comparison, Outcome, and Study design) criteria were also followed, as detailed below:

### 2.1 Literature searches

The search scope includes seven databases: PubMed, EMBASE, Cochrane Library, Web of Science (WOS), Chinese National Knowledge Infrastructure (CNKI), Wanfang Data Information Site, and VIP Information Database. The time range is from the establishment of the databases to November 21, 2024. The search strategy includes both subject headings and free terms, approximately as follows: (Stroke OR Brain Infarction OR Cerebral Infarction OR Cerebral Hemorrhage) AND (Medicine, Chinese Traditional) AND (Nursing). For the detailed search strategy, please refer to Supplementary material 2.

### 2.2 Criteria for inclusion and exclusion

Two researchers (YC and HL) independently screened eligible studies based on the inclusion and exclusion criteria. In case of disagreement, the two researchers resolved the issue through discussion.

Inclusion criteria:

1. Published, peer-reviewed journal articles with a RCT design, targeting stroke patients, written in Chinese or English.
2. The experimental group received one or more treatments from the 18 TCM nursing techniques, while the control group received conventional neurological nursing.
3. Outcome measures include one of the following: Fugl-Meyer Assessment (FMA), Modified Ashworth Scale (MAS), or Barthel Index (BI). The FMA (maximum score: 100) is a widely used and validated tool for evaluating motor recovery after stroke, particularly focusing on motor function of the upper and lower extremities; higher scores indicate better motor performance (8). The MAS is used to assess spasticity by evaluating the resistance encountered during passive muscle stretching, with scores ranging from 0 to 4, where higher scores reflect more severe spasticity (9). The BI (maximum score: 100) measures a patient’s ability to independently perform activities of daily living (ADLs), such as feeding, bathing, toileting, and mobility; higher scores represent greater functional independence (10).

Exclusion criteria:

1. Full text not available.
2. Inability to extract valid outcome measures.
3. Duplicate publications.

### 2.3 Data extraction

Two researchers (YC and HL) independently extracted the required information from the included studies and stored it in an Excel sheet. Disagreements were resolved through discussion, and missing information was obtained by contacting the authors. If there was no response, this was noted in the discussion section. The time point for outcome data was selected as the endpoint of the full treatment course. If there were multiple experimental groups, the group with the best therapeutic effect was chosen for the experimental group data.

Extract the following information:

1. First author and publication year;
2. Sample size (*n*);
3. Nursing interventions applied in the experimental group;
4. Nursing interventions applied in the control group;
5. Outcome measures (mean ± standard deviation);
6. Treatment duration.

### 2.4 Risk of bias assessment

Two researchers, YC and ZW, independently assessed the risk of bias of the included studies using the Cochrane Risk of Bias Tool 2 (RoB-2) (11). In cases of disagreement, a third reviewer (MJ) was consulted to reach a final decision.

### 2.5 GRADE level of evidence

Grades of Recommendation, Assessment, Development, and Evaluation (GRADE) is an internationally recognized system for assessing the quality of evidence and the strength of recommendations. Developed by the GRADE Working Group, it is widely used in systematic reviews, clinical practice guidelines, and health technology assessments (12). The GRADEpro GDT tool was used to evaluate the quality of evidence for three outcome indicators. Since all included studies were RCTs, the initial level of evidence was rated as High. The quality was then adjusted based on the following five downgrading factors: risk of bias, inconsistency, imprecision, indirectness, and publication bias. The final evidence levels were categorized as High, Moderate, Low, or Very Low.

### 2.6 Data analysis

The meta-analysis will be conducted using the *meta* package in R 4.4.2. At the end of the study, FMA and MAS will be used to measure limb motor function scores, and BI will be used to measure ADL scores. Standardized mean differences (SMD) will be used for summarizing the data. I^2^ will be used to quantify statistical heterogeneity, and when combining effect sizes, if the heterogeneity is less than 50%, a fixed-effects model will be used; if greater than 50%, a random-effects model will be applied. Funnel plots and Egger’s test will be used to detect publication bias, and if missing studies are identified, the trim-fill method will be applied to handle missing data. Sensitivity analysis will be performed to assess the robustness of the combined results. Meta-regression and subgroup analysis will be performed based on the following factors: hospital care vs. home care, hemorrhagic stroke vs. ischemic stroke, and high/medium/low risk of bias. A *p*-value of < 0.05 will be considered statistically significant.

## 3 Results

### 3.1 Study selection

**Figure 1:**
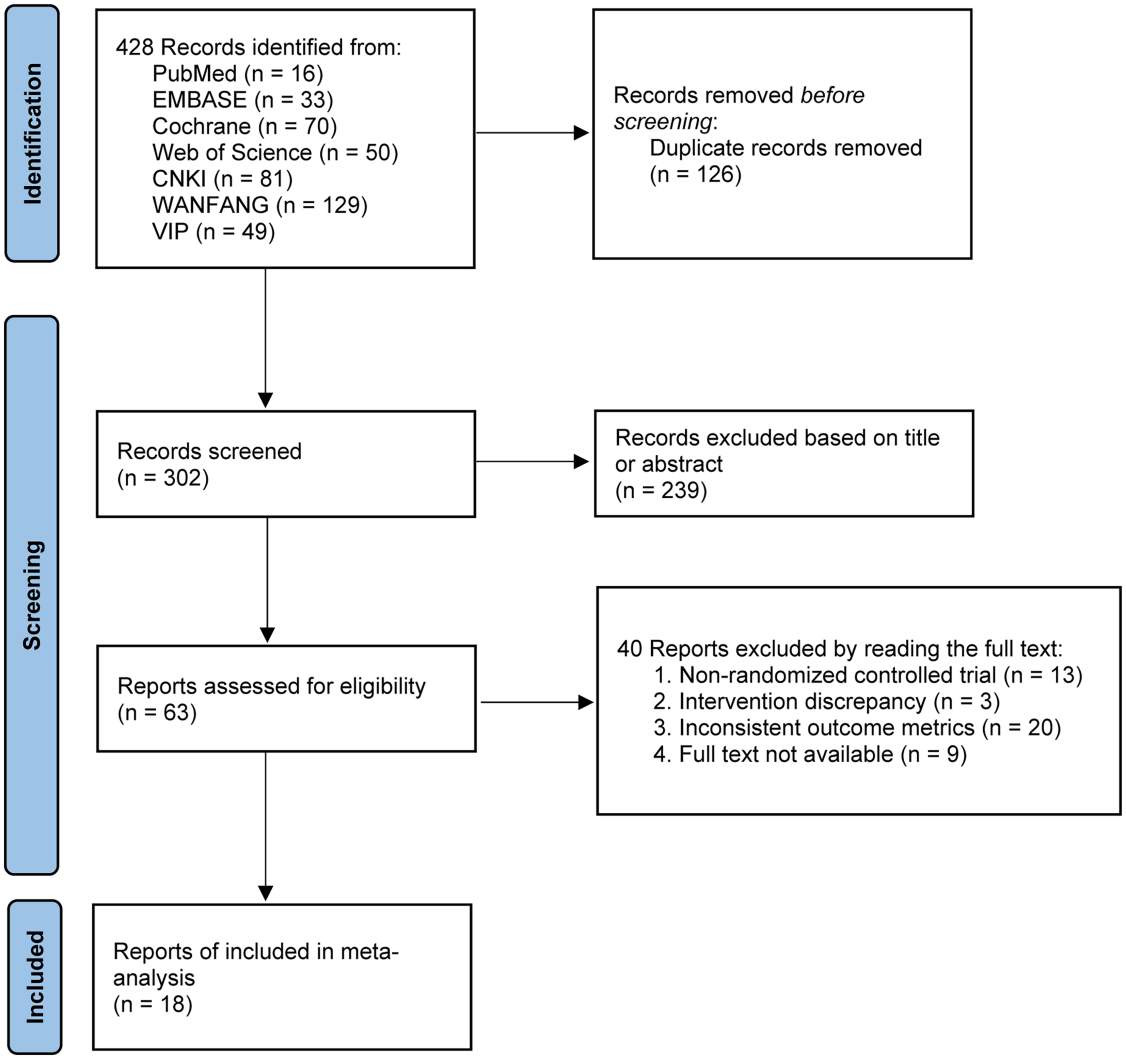
PRISMA 2020 flow diagram for literature screening.

Following the PRISMA guidelines and the predefined search strategy, a total of 428 studies were initially identified. After removing duplicates, 302 studies remained. Based on title and abstract screening, 239 studies were excluded. Among the remaining 63 full-text articles, 40 were excluded due to the following reasons: non-RCT (*n* = 13), inconsistent interventions (*n* = 3), outcome measures not aligned with inclusion criteria (*n* = 20), and full-text not accessible (*n* = 9). Ultimately, 18 studies were included in the meta-analysis (Figure 1).

### 3.2 Study characteristics

Among the 18 included studies (5–7, 13–27), various TCM nursing techniques were employed. Specifically, 13 studies applied acupoint massage, 4 used suspended moxibustion, 1 used herbal soaking, 4 used auricular point pressing, 4 applied acupoint application, 2 used herbal fumigation, 3 employed herbal hot compresses, 4 used cupping therapy, 1 applied herbal iontophoresis, 1 used wax therapy, and 1 employed herbal warm-moist compress. 16 studies adopted hospital-based nursing models, while 2 studies used home-based care models. Regarding stroke type, 6 studies focused on ischemic stroke, 1 on hemorrhagic stroke, and 11 on all types of stroke. In terms of outcomes, 8 studies reported the FMA, 5 reported the MAS, and 12 reported the BI. Detailed information is presented in Table 1.

**Table 1:** The information of the 18 included studies.

Author, year	Sample size (treatment/control)	Intervention measure	Control measure	Outcome	Course of treatment	Care setting	Stroke type	Risk of bias
Jin, 2021 (13)	48/46	Acupoint massage, suspended moxibustion, herbal soaking and conventional care	Conventional care	FMA	90 days	Home	Ischemic	Low
Liu, 2015 (14)	35/30	Acupoint massage and conventional care	Conventional care	FMA, BI	N/A	Hospital	All	Low
Song, 2017 (18)	30/30	Acupoint massage, acupoint application, auricular acupoint pressing and conventional care	Conventional care	FMA, BI	14 days	Hospital	Hemorrhagic	High
Wang1, 2016 (15)	40/40	Herbal fumigation and acupoint massage	Conventional care	FMA	N/A	Hospital	Ischemic	High
Wang, 2024 (17)	54/54	Acupoint massage, herbal hot compress and conventional care	Conventional care	FMA	90 days	Hospital	All	High
Zhu, 2012 (16)	28/28	Acupoint massage, cupping, suspended moxibustion and conventional care	Conventional care	FMA, BI	N/A	Hospital	All	High
Yue, 2013 (5)	35/34	Acupoint massage and conventional care	Conventional care	FMA, BI	90 days	Home	All	Low
Zhang, 2018 (19)	43/39	Cupping, scraping, herbal iontophoresis and conventional care	Conventional care	FMA, MAS, BI	60 days	Hospital	All	Some concerns
Li, 2019 (21)	38/39	Herbal hot compress and conventional care	Conventional care	MAS	28 days	Hospital	All	Low
Li, 2020 (23)	48/49	Herbal hot compress and conventional care	Conventional care	MAS	28 days	Hospital	All	Low
Li, 2022 (22)	42/45	Wax therapy and conventional care	Conventional care	MAS	28 days	Hospital	All	Low
Wang, 2023 (32)	39/39	Cupping, herbal warm-moist compress and conventional care	Herbal warm-moist compress and conventional care	MAS, BI	28 days	Hospital	All	Low
Jiang, 2009 (27)	36/36	Acupoint massage and conventional care	Conventional care	BI	40 days	Hospital	All	Low
Song, 2018 (7)	30/30	Acupoint massage, acupoint application, herbal fumigation, auricular acupoint pressing and conventional care	Conventional care	BI	14 days	Hospital	Ischemic	High
Tan, 2019 (26)	40/40	Acupoint massage, acupoint application, auricular acupoint pressing and conventional care	Conventional care	BI	N/A	Hospital	Ischemic	Low
Wang2, 2016 (24)	54/54	Acupoint massage, acupoint application, cupping, auricular acupoint pressing and suspended moxibustion conventional care	Conventional care	BI	63 days	Hospital	Ischemic	Low
Zhang, 2015 (25)	40/40	Acupoint massage and conventional care	Conventional care	BI	30 days	Hospital	Ischemic	Low
Guan, 2021 (6)	33/35	Acupoint massage, suspended moxibustion and conventional care	Conventional care	BI	28 days	Hospital	All	Low

FMA, Fugl-Meyer Assessment; MAS, Modified Ashworth Scale; BI, Barthel Index.

### 3.3 Risk of bias

**Figure 2:**
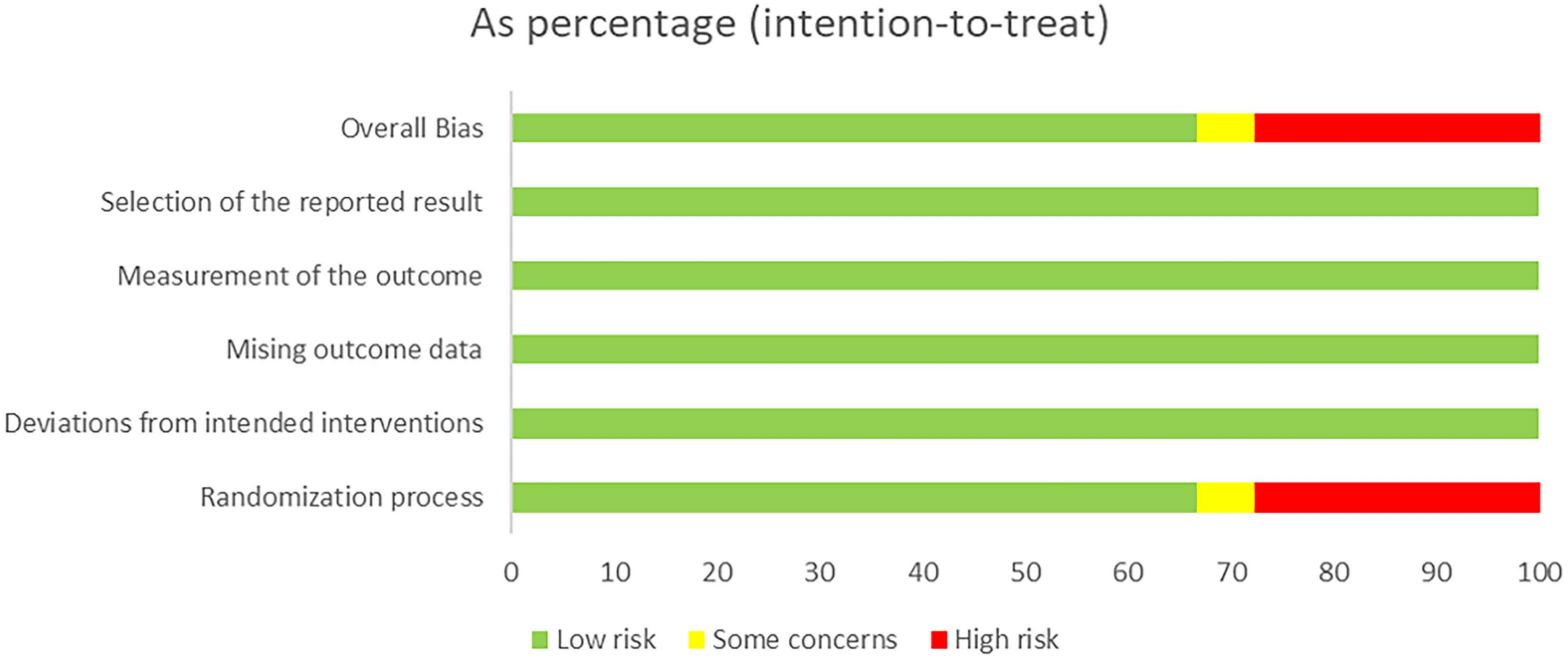
Risk of bias of the 18 studies.

Among the 18 included studies, 12 (66.67%) were rated as having a low risk of bias, 1 (5.56%) as having some concerns, and 5 (27.78%) as having a high risk of bias (Table 1). Specifically, 11 studies (61.11%) employed an appropriate method for random sequence generation, while only 2 studies (11.11%) clearly reported allocation concealment. None of the studies reported double-blinding of participants and personnel, and 5 studies (27.78%) mentioned blinding of outcome assessors. All studies showed no deviations from the intended interventions, no missing outcome data, and no selective reporting of results (Figure 2).

### 3.4 Efficacy of TCM nursing

**Figure 3:**
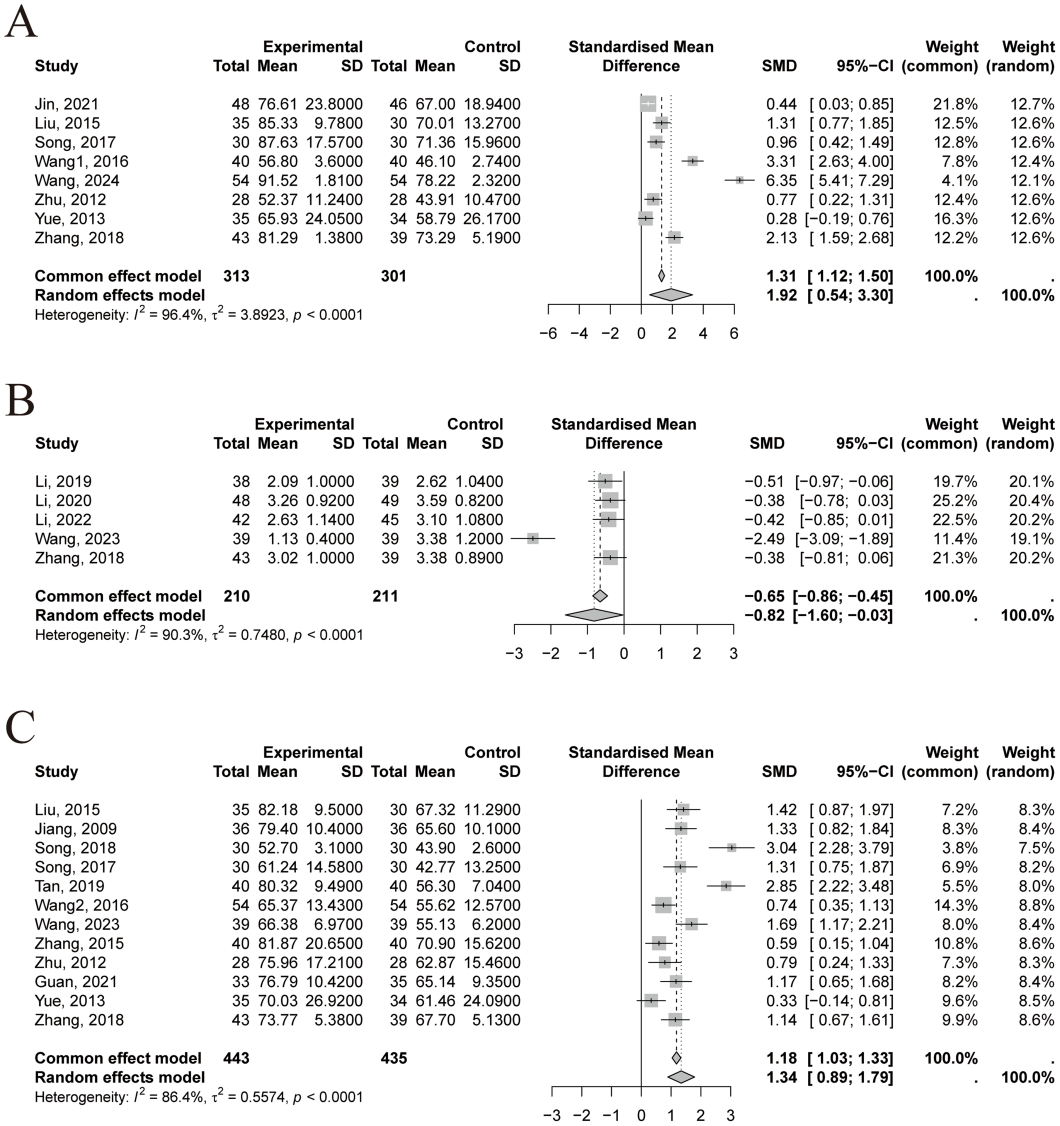
Summary statistics of effect sizes. **(A)** Fugl-Meyer Assessment. **(B)** Modified Ashworth Scale. **(C)** Barthel Index.

In terms of motor function, the pooled results from 8 studies demonstrated that TCM nursing interventions significantly improved FMA scores in stroke patients (*n* = 614, SMD = 1.92, 95% CI [0.54~3.30], *p* = 0.0066; heterogeneity: χ^2^ = 3.89, df = 7, *p* < 0.0001, I^2^ = 96.4%; Figure 3A). Similarly, 5 studies showed that TCM nursing interventions significantly improved MAS scores (*n* = 421, SMD = −0.82, 95% CI [−1.60~−0.03], *p* = 0.0416; heterogeneity: χ^2^ = 0.75, df = 4, *p* < 0.0001, I^2^ = 90.3%; Figure 3B). Regarding ADL, 12 studies revealed a significant improvement in BI scores following TCM nursing interventions (*n* = 878, SMD = 1.34, 95% CI [0.89~1.79], *p* < 0.0001; heterogeneity: χ^2^ = 0.56, df = 11, *p* < 0.0001, I^2^ = 86.4%; Figure 3C). For all three outcomes, I^2^ values exceeded 50%, indicating substantial heterogeneity. To explore the sources of heterogeneity, sensitivity analyses, meta-regression, and subgroup analyses were subsequently conducted.

### 3.5 Publication bias

**Figure 4:**
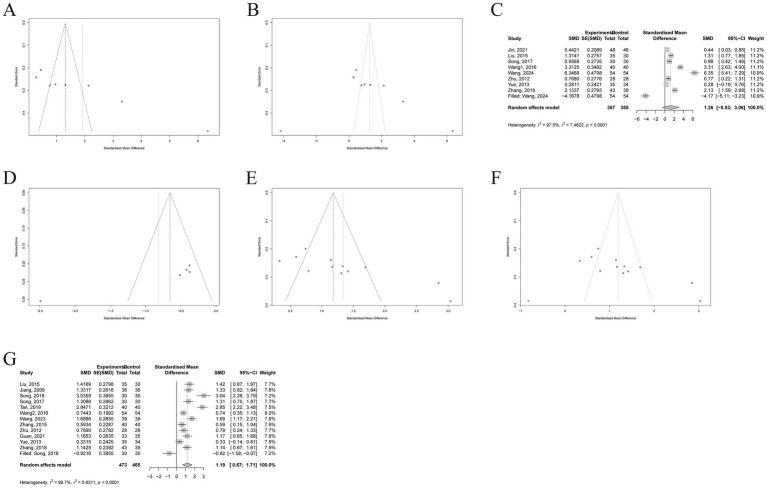
Publication bias. **(A)** Funnel plot of FMA. **(B)** Funnel plot after trim-fill of FMA, **(C)** Forest plot after trim-fill of FMA. **(D)** Funnel plot of MAS. **(E)** Funnel plot of BI. **(F)** Funnel plot after trim-fill of BI. **(G)** Forest plot after trim-fill of BI. FMA, Fugl-Meyer Assessment; MAS, Modified Ashworth Scale; BI, Barthel Index.

Funnel plots and Egger’s tests were employed to assess potential publication bias. The funnel plot for FMA showed asymmetry (Figure 4A), and the Egger’s test indicated significant publication bias (*p* = 0.0006). After applying the trim-fill method, one study was imputed, resulting in a corrected effect size that differed from the original (SMD = 1.26, 95% CI [−0.53~3.06], *p* = 0.1682; Figures 4B,C). For MAS, the funnel plot also exhibited asymmetry (Figure 4D), and the Egger’s test yielded a *p*-value of 0.0085. However, the trim-fill analysis indicated no missing studies. Regarding BI, the funnel plot appeared asymmetric (Figure 4E), with an Egger’s test p-value of 0.0013. One study was imputed using the trim-fill method, and the adjusted effect size remained consistent with the original result (SMD = 1.19, 95% CI [0.67~1.71], *p* < 0.0001; Figures 4F,G). The above results suggest that the meta-analysis findings for FMA, MAS, and BI may be subject to publication bias. After controlling for publication bias using the trim-fill method, MAS and BI still showed significant differences, indicating that their results are relatively robust. However, for FMA, the results were no longer significant after adjusting for publication bias, which suggests that the initially observed significant difference may have been due to bias.

### 3.6 Sensitivity analysis

**Figure 5:**
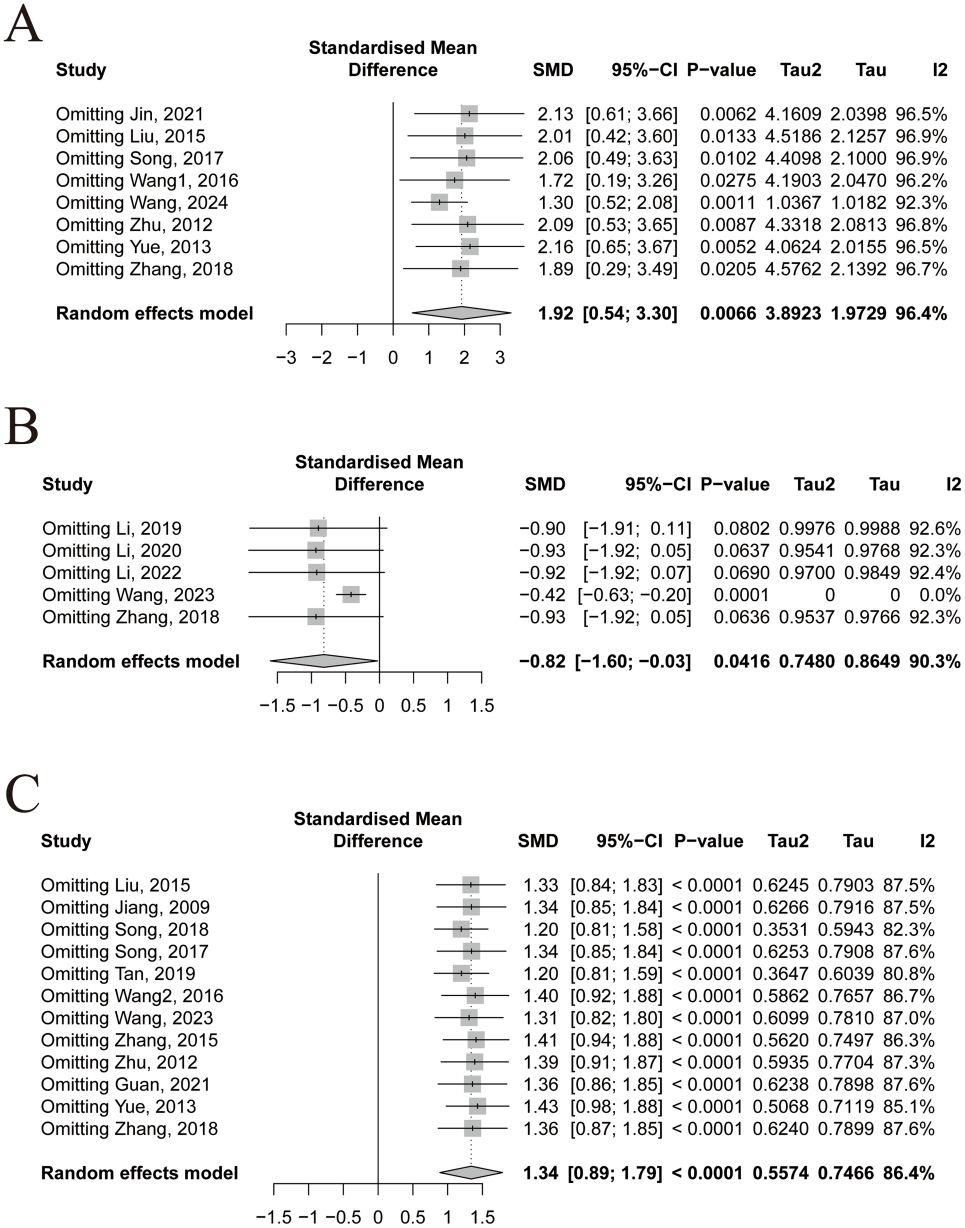
Sensitivity analysis. **(A)** Fugl-Meyer Assessment. **(B)** Modified Ashworth Scale. **(C)** Barthel Index.

Sensitivity analysis showed that the combined effect sizes for FMA and BI remained statistically significant after excluding any single study (Figures 5A,C), indicating good robustness of the meta-analysis results and suggesting that the observed heterogeneity was not driven by any individual study. For MAS, heterogeneity dropped to I^2^ = 0% after excluding the study by Wang (20), suggesting that this study was the primary source of heterogeneity. Furthermore, upon excluding this study, the effect remained statistically significant (*p* = 0.0001), whereas exclusion of any other single study resulted in non-significant *p*-values (all *p* > 0.05; Figure 5B). These findings indicate that the MAS-related results are not stable and highlight the need for additional large-scale studies to provide more reliable evidence for clinical practice.

### 3.7 Meta-regression and subgroup analysis

**Figure 6:**
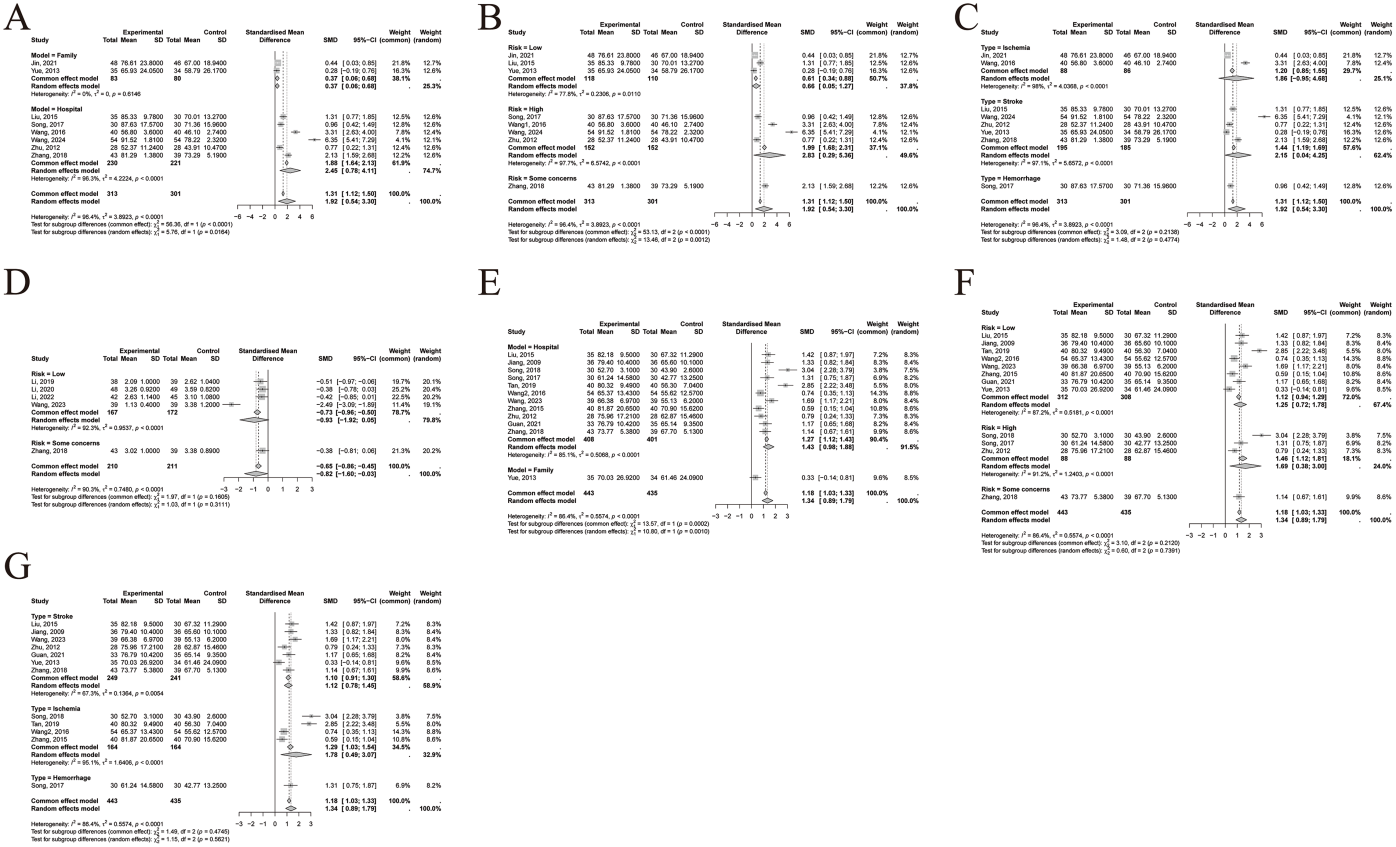
Subgroup analysis. **(A)** Care setting of FMA. **(B)** Risk of bias of FMA. **(C)** Stroke type of FMA. **(D)** Risk of bias of MAS. **(E)** Care setting of BI. **(F)** Risk of bias of BI. **(G)** Stroke type of BI. FMA, Fugl-Meyer Assessment; MAS, Modified Ashworth Scale; BI, Barthel Index.

Meta-regression and subgroup analyses were conducted to explore potential sources of heterogeneity based on care setting (hospital-based vs. home-based), stroke type (ischemic vs. hemorrhagic), and risk of bias (high, unclear, or low). Meta-regression results indicated that none of these factors significantly contributed to the heterogeneity in any of the outcome measures (all *p* > 0.05). Subgroup analyses further confirmed these findings, as within-group heterogeneity remained high (I^2^ > 50%) across all subgroups, suggesting that these factors were not the primary sources of heterogeneity. However, some notable differences in effect sizes were observed between subgroups:

1 Fugl-Meyer Assessment (FMA):

A significant difference was found between care settings (χ^2^ = 5.76, df = 1, *p* = 0.0164), with hospital-based care (SMD = 2.45, 95% CI [0.78~4.11]) showing greater improvements than home-based care (SMD = 0.37, 95% CI [0.06~0.68]; Figure 6A). Risk of bias also influenced the effect size significantly (χ^2^ = 13.46, df = 2, *p* = 0.0012), with studies at high risk (SMD = 2.83, 95% CI [0.29~5.36]) and unclear risk (SMD = 2.13, 95% CI [1.59 ~ 2.68]) reporting larger effects than those at low risk (SMD = 0.66, 95% CI [0.05~1.27]; Figure 6B). No significant difference was observed across stroke types (χ^2^ = 1.48, df = 2, *p* = 0.4774; Figure 6C).

2 Modified Ashworth Scale (MAS):

No significant difference in effect size was found among different risk of bias levels (χ^2^ = 1.03, df = 1, *p* = 0.3111; Figure 6D).

3 Barthel Index (BI):

A significant difference was observed between care settings (χ^2^ = 10.80, df = 1, *p* = 0.0010), with hospital-based care (SMD = 1.43, 95% CI [0.98~1.88]) being more effective than home-based care (SMD = 0.33, 95% CI [−0.14~0.81]; Figure 6E). No significant differences were found across different levels of risk of bias (χ^2^ = 0.60, df = 2, *p* = 0.7391; Figure 6F) or stroke types (χ^2^ = 1.15, df = 2, *p* = 0.5621; Figure 6G).

### 3.8 GRADE evidence assessment

**Figure 7:**
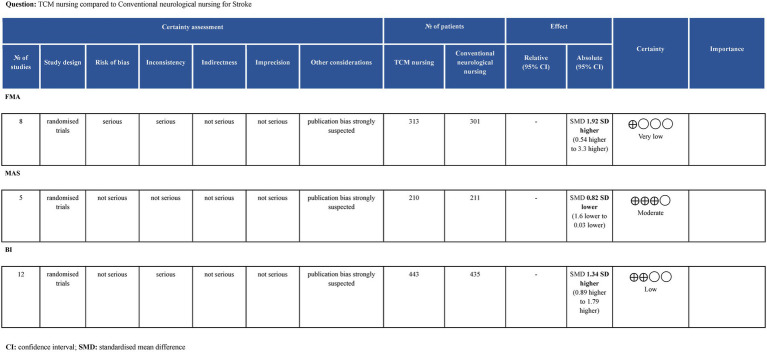
GRADE level of evidence.

The evaluation results show that the Egger’s test for all three outcome indicators exhibited significant differences, leading to the assessment of “publication bias strongly suspected.” For FMA, the RoB-2 assessment revealed that half of the studies had a risk of bias, with the heterogeneity test showing I^2^ > 50% and no identifiable source of heterogeneity. As a result, both “Risk of bias” and “Inconsistency” were rated as “serious,” leading to an overall evidence quality of Very Low. For MAS, although the heterogeneity test (I^2^ > 50%) indicated high heterogeneity, sensitivity analysis identified the source of heterogeneity (20), so “Inconsistency” was rated as “not serious,” resulting in an overall evidence quality of Moderate. For BI, due to the heterogeneity test (I^2^ > 50%) showing high heterogeneity and no identified sources of heterogeneity, “Inconsistency” was rated as “serious,” resulting in an overall evidence quality of Low (Figure 7).

## 4 Discussion

### 4.1 Summary of evidence

Hu et al. (28) conducted an umbrella review suggesting that TCM nursing may be effective for stroke management; however, the credibility of the findings was limited. Notably, their study was based on the *Technical Operation Regulations of Traditional Chinese Nursing Practices* issued by the Chinese Association of Traditional Chinese Medicine in 2006—a guideline that is now outdated and lacks contemporary clinical relevance. In contrast, the *National Administration of Traditional Chinese Medicine* introduced an updated set of 18 standardized TCM nursing techniques in 2015. These techniques span a broad spectrum, from external herbal applications to modern acupoint-based interventions, and are more representative of current TCM nursing practices. To build upon and update existing evidence, the present study systematically synthesized all available literature to date on TCM nursing interventions for stroke. However, due to the limited number of studies using the same nursing technique, which did not support a meta-analysis, we combined different TCM nursing techniques for a unified analysis. The meta-analysis revealed that TCM nursing significantly improved motor function and ADL in stroke patients, as measured by FMA (SMD = 1.92, *p* = 0.0066), MAS (SMD = −0.82, *p* = 0.0416), and BI (SMD = 1.34, *p* < 0.0001). However, these aggregated effect sizes were accompanied by substantial heterogeneity and potential publication bias. To address these concerns, we conducted sensitivity analyses, meta-regression, and subgroup analyses to explore the sources of heterogeneity, and used Egger’s test along with the trim-fill method to assess and adjust for possible publication bias.

### 4.2 Risk of bias

The Cochrane risk of bias assessment showed that, regarding randomization, more than half of the studies reported random allocation methods, while only a small number mentioned allocation concealment. Nonetheless, scientifically appropriate randomization methods—such as computer-generated sequences or random number tables—can prevent investigators or participants from knowing the group assignments in advance. Therefore, studies that reported proper randomization methods were considered to have a low risk of bias in the randomization process.

In terms of blinding, none of the studies reported blinding of participants or personnel. In drug trials, placebo use is the preferred approach to achieve double-blinding; however, for interventional therapies, it is often difficult to blind the operator. Certain traditional Chinese nursing techniques, such as acupoint application or external herbal treatments, may allow double-blinding through the use of identical-appearing and similarly scented preparations. In contrast, techniques like massage, moxibustion, and auricular point pressing are challenging to blind from the practitioner. Although both the operators and participants might have been aware of the treatment, given that all studies showed no deviation from the intended interventions, this domain was judged as low risk. Additionally, only a few studies reported blinding of outcome assessors. However, considering that the outcome measures were not based on subjective perception (e.g., pain scores), but rather were relatively objective, the risk of bias in outcome measurement was considered low. Finally, none of the studies had missing outcome data or selectively reported results. In summary, the overall risk of bias in the 18 included studies was low, with the primary source of high risk stemming from inadequate randomization methods.

In terms of reporting bias, Egger’s test suggested potential publication bias across all three outcome indicators. After applying the trim-fill method, the summary effect sizes for MAS and BI remained robust, while the result for FMA was no longer significant after controlling for publication bias. This suggests that the initially observed significant difference for FMA may have been due to bias. These results indicate that TCM nursing techniques are more effective in improving spasticity and ADL in stroke patients, whereas their effect on upper and lower limb motor function recovery remains debatable.

### 4.3 Sources of heterogeneity

All three pooled outcomes exhibited I^2^ values greater than 50%, indicating substantial heterogeneity. To investigate its sources, we conducted sensitivity analysis, meta-regression, and subgroup analyses. Sensitivity analysis showed that for MAS, heterogeneity disappeared (I^2^ = 0%) after excluding the study by Wang (20), suggesting that this study was the primary source of heterogeneity. Upon re-examination, we found that the control group in this study received conventional care combined with herbal hot compress, whereas other studies’ control groups received conventional care alone, which may partly explain the observed heterogeneity. For FMA and BI, excluding any single study did not significantly alter heterogeneity, indicating that heterogeneity was not driven by an individual study. Meta-regression analyses showed that care setting, stroke type, and risk of bias were not significant sources of heterogeneity for any of the outcomes (all *p* > 0.05). Subgroup analyses further demonstrated that within-group heterogeneity remained high (I^2^ > 50%), suggesting that these factors could not explain the heterogeneity either.

We speculate that part of the heterogeneity may stem from differences in the specific nursing techniques applied in the intervention groups. Across the included studies, various combinations of 18 TCM nursing techniques were used, which could contribute to inconsistency. Using identical nursing techniques might reduce between-study heterogeneity; however, currently, there are insufficient RCTs focusing on the same technique to allow for separate meta-analysis. In addition, heterogeneity may also stem from inconsistencies in outcome assessment timing, with time spans ranging from 2 weeks to 3 months post-treatment. Since 4 studies did not provide specific time points for outcome assessments, we did not conduct a subgroup analysis (14–16, 26). Considering the above, we employed a random-effects model to obtain relatively reliable results despite the presence of heterogeneity.

### 4.4 Subgroup analysis

Subgroup analyses based on study characteristics revealed additional findings beyond heterogeneity exploration. Both hospital-based and home-based care models significantly improved limb motor function and ADL in stroke patients, with hospital-based care showing greater improvements. Home-based care is an important component of nursing practice. A bibliometric analysis based on the WOS indicated that the number of publications on home-based care has been steadily increasing, with “quality of life” identified as a major research focus (29). However, home-based care is still at an early stage of development and remains relatively underutilized in clinical practice; for example, among the 18 included studies, only two adopted a home-based care model (5, 13). Moreover, current research in this area remains relatively narrow in scope, lacking interdisciplinary integration, which warrants further exploration and refinement in future practice. In addition, subgroup analyses revealed no significant differences in outcome measures across different types of stroke, suggesting that Chinese medicine nursing techniques are similarly effective for both hemorrhagic and ischemic stroke patients. Finally, studies with a higher risk of bias reported larger effect sizes, highlighting the need for cautious interpretation of results and emphasizing the importance of more rigorous study designs in future research.

### 4.5 Interpretation of evidence quality

The GRADE results indicate that FMA was rated as Very Low, meaning there is almost no confidence in the effect estimate: the true value is likely to differ significantly from the estimated value. MAS was rated as Moderate, suggesting moderate confidence in the effect estimate: the true value may be close to the estimate, but there is still a possibility that they could differ substantially. BI was rated as Low, indicating limited confidence in the effect estimate: the true value could differ significantly from the estimated value. The main downgrading factors were publication bias, risk of bias, and heterogeneity. Future studies should aim to improve in terms of multicenter design, large sample sizes, and rigorous trial methodology.

### 4.6 Potential mechanisms of TCM on stroke

Several animal studies have begun to elucidate the biological mechanisms underlying the effects of commonly used TCM nursing techniques for stroke treatment. Research has shown that moxibustion can reduce lipid peroxidation levels after cerebral ischemia–reperfusion, decrease reactive oxygen species accumulation, inhibit ferroptosis of neuronal cells, and alleviate neurological impairment (30). Other studies have demonstrated that acupoint application can lower the expression of inflammatory cytokines following ischemia–reperfusion injury, exert anti-apoptotic effects, significantly reduce infarct volume, and improve neurological deficits (31). Additionally, auricular acupressure has been found to ameliorate cerebral ischemic injury, potentially by regulating immune-inflammatory responses (32). However, animal studies on other TCM nursing techniques remain limited, possibly due to the technical challenges of replicating interventions such as acupoint massage, cupping, and scraping therapy in animal models. This area warrants further exploration.

Based on the available literature, inflammatory response appears to be a key mechanism through which TCM nursing techniques exert therapeutic effects in stroke. Current studies mainly focus on changes in inflammatory cytokines; future research could delve deeper into upstream mechanisms, such as microglial phenotype modulation and pyroptosis, to better understand the underlying processes.

### 4.7 Strengths and limitations

Based on the latest guidelines issued by the *National Administration of Traditional Chinese Medicine*, this study systematically retrieved RCTs from seven major databases to evaluate the effects of TCM nursing techniques on motor function and ADL in stroke patients—an effort that represents the first of its kind both domestically and internationally. Furthermore, given the considerable heterogeneity among the included studies, we employed three approaches—sensitivity analysis, meta-regression, and subgroup analysis—to explore potential sources of heterogeneity. Nevertheless, there is still high heterogeneity within the subgroups, which may be related to differences in the TCM nursing techniques and outcome assessment time points across studies. Additionally, the lack of allocation concealment and blinding reporting in most studies indirectly led to the poor GRADE assessments for FMA and BI. Therefore, the above results should be interpreted with caution.

### 4.8 Recommendations for future research

During the course of this study, we identified several issues in the current TCM nursing technique-related RCTs, such as inconsistent interventions and the lack of blinding. We recommend that future RCTs adopt more uniform intervention protocols to reduce heterogeneity, which would also be beneficial for the development of clinical guidelines or industry standards. Additionally, some TCM nursing techniques, such as acupoint application and external use of Chinese medicine, are suitable for blinding with placebos, which can be achieved using visually and olfactorily similar substances.

## Data Availability

The original contributions presented in the study are included in the article/Supplementary material, further inquiries can be directed to the corresponding authors.
