# Supplementary material for: Efficacy of traditional Chinese medicine nursing in stroke: a systematic review and meta-analysis
Source: Front Neurol. 2025 Aug 12;16:1657802. doi: 10.3389/fneur.2025.1657802 (PMC12378047; doi:10.3389/fneur.2025.1657802)
Supplement: Supplementary file 2 [file Table_2.docx]

**Search strategy**

# PubMed

((“Stroke”[MeSH]) OR (“Brain Infarction”[MeSH]) OR (“Cerebral Infarction”[MeSH]) OR (“Cerebral Hemorrhage”[MeSH]) OR (Stroke[Title/Abstract]) OR (Strokes[Title/Abstract]) OR (Cerebrovascular Accident[Title/Abstract]) OR (Cerebrovascular Accidents[Title/Abstract]) OR (Cerebral Stroke[Title/Abstract]) OR (Cerebral Strokes[Title/Abstract]) OR (Stroke, Cerebral[Title/Abstract]) OR (Strokes, Cerebral[Title/Abstract]) OR (Cerebrovascular Apoplexy[Title/Abstract]) OR (Apoplexy, Cerebrovascular[Title/Abstract]) OR (Vascular Accident, Brain[Title/Abstract]) OR (Brain Vascular Accident[Title/Abstract]) OR (Brain Vascular Accidents[Title/Abstract]) OR (Vascular Accidents, Brain[Title/Abstract]) OR (Cerebrovascular Stroke[Title/Abstract]) OR (Cerebrovascular Strokes[Title/Abstract]) OR (Stroke, Cerebrovascular[Title/Abstract]) OR (Strokes, Cerebrovascular[Title/Abstract]) OR (Apoplexy[Title/Abstract]) OR (CVA (Cerebrovascular Accident[Title/Abstract])) OR (CVAs (Cerebrovascular Accident[Title/Abstract])) OR (Stroke, Acute[Title/Abstract]) OR (Acute Stroke[Title/Abstract]) OR (Acute Strokes[Title/Abstract]) OR (Strokes, Acute[Title/Abstract]) OR (Cerebrovascular Accident, Acute[Title/Abstract]) OR (Acute Cerebrovascular Accident[Title/Abstract]) OR (Acute Cerebrovascular Accidents[Title/Abstract]) OR (Cerebrovascular Accidents, Acute[Title/Abstract]) OR (Brain Infarction[Title/Abstract]) OR (Brain Infarctions[Title/Abstract]) OR (Infarction, Brain[Title/Abstract]) OR (Infarctions, Brain[Title/Abstract]) OR (Brain Infarct[Title/Abstract]) OR (Brain Infarcts[Title/Abstract]) OR (Infarct, Brain[Title/Abstract]) OR (Infarcts, Brain[Title/Abstract]) OR (Venous Infarction, Brain[Title/Abstract]) OR (Brain Venous Infarction[Title/Abstract]) OR (Brain Venous Infarctions[Title/Abstract]) OR (Infarction, Brain Venous[Title/Abstract]) OR (Infarctions, Brain Venous[Title/Abstract]) OR (Venous Infarctions, Brain[Title/Abstract]) OR (Brain Infarction, Venous[Title/Abstract]) OR (Brain Infarctions, Venous[Title/Abstract]) OR (Infarctions, Venous Brain[Title/Abstract]) OR (Infarction, Venous Brain[Title/Abstract]) OR (Venous Brain Infarction[Title/Abstract]) OR (Venous Brain Infarctions[Title/Abstract]) OR (Anterior Cerebral Circulation Infarction[Title/Abstract]) OR (Infarction, Anterior Cerebral Circulation[Title/Abstract]) OR (Anterior Circulation Brain Infarction[Title/Abstract]) OR (Anterior Circulation Infarction, Brain[Title/Abstract]) OR (Brain Infarction, Anterior Circulation[Title/Abstract]) OR (Infarction, Anterior Circulation, Brain[Title/Abstract]) OR (Infarction, Brain, Anterior Circulation[Title/Abstract]) OR (Brain Infarction, Posterior Circulation[Title/Abstract]) OR (Posterior Circulation Brain Infarction[Title/Abstract]) OR (Posterior Circulation Infarction, Brain[Title/Abstract]) OR (Infarction, Brain, Posterior Circulation[Title/Abstract]) OR (Infarction, Posterior Circulation, Brain[Title/Abstract]) OR (Cerebral Infarction[Title/Abstract]) OR (Cerebral Infarctions[Title/Abstract]) OR (Infarctions, Cerebral[Title/Abstract]) OR (Cerebral Infarct[Title/Abstract]) OR (Cerebral Infarcts[Title/Abstract]) OR (Infarct, Cerebral[Title/Abstract]) OR (Infarcts, Cerebral[Title/Abstract]) OR (Infarction, Cerebral[Title/Abstract]) OR (Anterior Choroidal Artery Infarction[Title/Abstract]) OR (Posterior Choroidal Artery Infarction[Title/Abstract]) OR (Subcortical Infarction[Title/Abstract]) OR (Infarctions, Subcortical[Title/Abstract]) OR (Infarction, Subcortical[Title/Abstract]) OR (Subcortical Infarctions[Title/Abstract]) OR (Cerebral Infarction, Left Hemisphere[Title/Abstract]) OR (Cerebral, Left Hemisphere, Infarction[Title/Abstract]) OR (Infarction, Cerebral, Left Hemisphere[Title/Abstract]) OR (Left Hemisphere, Infarction, Cerebral[Title/Abstract]) OR (Infarction, Left Hemisphere, Cerebral[Title/Abstract]) OR (Left Hemisphere, Cerebral Infarction[Title/Abstract]) OR (Cerebral Infarction, Right Hemisphere[Title/Abstract]) OR (Cerebral, Right Hemisphere, Infarction[Title/Abstract]) OR (Infarction, Cerebral, Right Hemisphere[Title/Abstract]) OR (Infarction, Right Hemisphere, Cerebral[Title/Abstract]) OR (Right Hemisphere, Infarction, Cerebral[Title/Abstract]) OR (Right Hemisphere, Cerebral Infarction[Title/Abstract]) OR (Cerebral Hemorrhage[Title/Abstract]) OR (Hemorrhage, Cerebral[Title/Abstract]) OR (Cerebral Hemorrhages[Title/Abstract]) OR (Hemorrhages, Cerebral[Title/Abstract]) OR (Intracerebral Hemorrhage[Title/Abstract]) OR (Hemorrhage, Intracerebral[Title/Abstract]) OR (Hemorrhages, Intracerebral[Title/Abstract]) OR (Intracerebral Hemorrhages[Title/Abstract]) OR (Hemorrhage, Cerebrum[Title/Abstract]) OR (Cerebrum Hemorrhage[Title/Abstract]) OR (Cerebrum Hemorrhages[Title/Abstract]) OR (Hemorrhages, Cerebrum[Title/Abstract]) OR (Brain Hemorrhage, Cerebral[Title/Abstract]) OR (Brain Hemorrhages, Cerebral[Title/Abstract]) OR (Cerebral Brain Hemorrhage[Title/Abstract]) OR (Cerebral Brain Hemorrhages[Title/Abstract]) OR (Hemorrhage, Cerebral Brain[Title/Abstract]) OR (Hemorrhages, Cerebral Brain[Title/Abstract]) OR (Cerebral Parenchymal Hemorrhage[Title/Abstract]) OR (Cerebral Parenchymal Hemorrhages[Title/Abstract]) OR (Hemorrhage, Cerebral Parenchymal[Title/Abstract]) OR (Hemorrhages, Cerebral Parenchymal[Title/Abstract]) OR (Parenchymal Hemorrhage, Cerebral[Title/Abstract]) OR (Parenchymal Hemorrhages, Cerebral[Title/Abstract])) AND ((“Medicine, Chinese Traditional”[MeSH]) OR (Medicine, Chinese Traditional[Title/Abstract]) OR (Zhong Yi Xue[Title/Abstract]) OR (Chung I Hsueh[Title/Abstract]) OR (Hsueh, Chung I[Title/Abstract]) OR (Traditional Medicine, Chinese[Title/Abstract]) OR (Chinese Traditional Medicine[Title/Abstract]) OR (Traditional Chinese Medicine[Title/Abstract]) OR (Chinese Medicine, Traditional[Title/Abstract]) OR (Traditional Tongue Diagnosis[Title/Abstract]) OR (Tongue Diagnoses, Traditional[Title/Abstract]) OR (Tongue Diagnosis, Traditional[Title/Abstract]) OR (Traditional Tongue Diagnoses[Title/Abstract]) OR (Traditional Tongue Assessment[Title/Abstract]) OR (Tongue Assessment, Traditional[Title/Abstract]) OR (Traditional Tongue Assessments[Title/Abstract]) OR (TCM[Title/Abstract]) OR (Chinese Medicine[Title/Abstract])) AND ((“Nursing”[MeSH]) OR (Nursing[Title/Abstract]) OR (Nursings[Title/Abstract]))

# Embase

('Stroke’:ab,ti OR ‘Strokes’:ab,ti OR ‘Cerebrovascular Accident’:ab,ti OR ‘Cerebrovascular Accidents’:ab,ti OR ‘Cerebral Stroke’:ab,ti OR ‘Cerebral Strokes’:ab,ti OR ‘Stroke, Cerebral’:ab,ti OR ‘Strokes, Cerebral’:ab,ti OR ‘Cerebrovascular Apoplexy’:ab,ti OR ‘Apoplexy, Cerebrovascular’:ab,ti OR ‘Vascular Accident, Brain’:ab,ti OR ‘Brain Vascular Accident’:ab,ti OR ‘Brain Vascular Accidents’:ab,ti OR ‘Vascular Accidents, Brain’:ab,ti OR ‘Cerebrovascular Stroke’:ab,ti OR ‘Cerebrovascular Strokes’:ab,ti OR ‘Stroke, Cerebrovascular’:ab,ti OR ‘Strokes, Cerebrovascular’:ab,ti OR ‘Apoplexy’:ab,ti OR ‘CVA (Cerebrovascular Accident)’:ab,ti OR ‘CVAs (Cerebrovascular Accident)’:ab,ti OR ‘Stroke, Acute’:ab,ti OR ‘Acute Stroke’:ab,ti OR ‘Acute Strokes’:ab,ti OR ‘Strokes, Acute’:ab,ti OR ‘Cerebrovascular Accident, Acute’:ab,ti OR ‘Acute Cerebrovascular Accident’:ab,ti OR ‘Acute Cerebrovascular Accidents’:ab,ti OR ‘Cerebrovascular Accidents, Acute’:ab,ti OR ‘Brain Infarction’:ab,ti OR ‘Brain Infarctions’:ab,ti OR ‘Infarction, Brain’:ab,ti OR ‘Infarctions, Brain’:ab,ti OR ‘Brain Infarct’:ab,ti OR ‘Brain Infarcts’:ab,ti OR ‘Infarct, Brain’:ab,ti OR ‘Infarcts, Brain’:ab,ti OR ‘Venous Infarction, Brain’:ab,ti OR ‘Brain Venous Infarction’:ab,ti OR ‘Brain Venous Infarctions’:ab,ti OR ‘Infarction, Brain Venous’:ab,ti OR ‘Infarctions, Brain Venous’:ab,ti OR ‘Venous Infarctions, Brain’:ab,ti OR ‘Brain Infarction, Venous’:ab,ti OR ‘Brain Infarctions, Venous’:ab,ti OR ‘Infarctions, Venous Brain’:ab,ti OR ‘Infarction, Venous Brain’:ab,ti OR ‘Venous Brain Infarction’:ab,ti OR ‘Venous Brain Infarctions’:ab,ti OR ‘Anterior Cerebral Circulation Infarction’:ab,ti OR ‘Infarction, Anterior Cerebral Circulation’:ab,ti OR ‘Anterior Circulation Brain Infarction’:ab,ti OR ‘Anterior Circulation Infarction, Brain’:ab,ti OR ‘Brain Infarction, Anterior Circulation’:ab,ti OR ‘Infarction, Anterior Circulation, Brain’:ab,ti OR ‘Infarction, Brain, Anterior Circulation’:ab,ti OR ‘Brain Infarction, Posterior Circulation’:ab,ti OR ‘Posterior Circulation Brain Infarction’:ab,ti OR ‘Posterior Circulation Infarction, Brain’:ab,ti OR ‘Infarction, Brain, Posterior Circulation’:ab,ti OR ‘Infarction, Posterior Circulation, Brain’:ab,ti OR ‘Cerebral Infarction’:ab,ti OR ‘Cerebral Infarctions’:ab,ti OR ‘Infarctions, Cerebral’:ab,ti OR ‘Cerebral Infarct’:ab,ti OR ‘Cerebral Infarcts’:ab,ti OR ‘Infarct, Cerebral’:ab,ti OR ‘Infarcts, Cerebral’:ab,ti OR ‘Infarction, Cerebral’:ab,ti OR ‘Anterior Choroidal Artery Infarction’:ab,ti OR ‘Posterior Choroidal Artery Infarction’:ab,ti OR ‘Subcortical Infarction’:ab,ti OR ‘Infarctions, Subcortical’:ab,ti OR ‘Infarction, Subcortical’:ab,ti OR ‘Subcortical Infarctions’:ab,ti OR ‘Cerebral Infarction, Left Hemisphere’:ab,ti OR ‘Cerebral, Left Hemisphere, Infarction’:ab,ti OR ‘Infarction, Cerebral, Left Hemisphere’:ab,ti OR ‘Left Hemisphere, Infarction, Cerebral’:ab,ti OR ‘Infarction, Left Hemisphere, Cerebral’:ab,ti OR ‘Left Hemisphere, Cerebral Infarction’:ab,ti OR ‘Cerebral Infarction, Right Hemisphere’:ab,ti OR ‘Cerebral, Right Hemisphere, Infarction’:ab,ti OR ‘Infarction, Cerebral, Right Hemisphere’:ab,ti OR ‘Infarction, Right Hemisphere, Cerebral’:ab,ti OR ‘Right Hemisphere, Infarction, Cerebral’:ab,ti OR ‘Right Hemisphere, Cerebral Infarction’:ab,ti OR ‘Cerebral Hemorrhage’:ab,ti OR ‘Hemorrhage, Cerebral’:ab,ti OR ‘Cerebral Hemorrhages’:ab,ti OR ‘Hemorrhages, Cerebral’:ab,ti OR ‘Intracerebral Hemorrhage’:ab,ti OR ‘Hemorrhage, Intracerebral’:ab,ti OR ‘Hemorrhages, Intracerebral’:ab,ti OR ‘Intracerebral Hemorrhages’:ab,ti OR ‘Hemorrhage, Cerebrum’:ab,ti OR ‘Cerebrum Hemorrhage’:ab,ti OR ‘Cerebrum Hemorrhages’:ab,ti OR ‘Hemorrhages, Cerebrum’:ab,ti OR ‘Brain Hemorrhage, Cerebral’:ab,ti OR ‘Brain Hemorrhages, Cerebral’:ab,ti OR ‘Cerebral Brain Hemorrhage’:ab,ti OR ‘Cerebral Brain Hemorrhages’:ab,ti OR ‘Hemorrhage, Cerebral Brain’:ab,ti OR ‘Hemorrhages, Cerebral Brain’:ab,ti OR ‘Cerebral Parenchymal Hemorrhage’:ab,ti OR ‘Cerebral Parenchymal Hemorrhages’:ab,ti OR ‘Hemorrhage, Cerebral Parenchymal’:ab,ti OR ‘Hemorrhages, Cerebral Parenchymal’:ab,ti OR ‘Parenchymal Hemorrhage, Cerebral’:ab,ti OR ‘Parenchymal Hemorrhages, Cerebral’:ab,ti) AND ('Medicine, Chinese Traditional’:ab,ti OR ‘Zhong Yi Xue’:ab,ti OR ‘Chung I Hsueh’:ab,ti OR ‘Hsueh, Chung I’:ab,ti OR ‘Traditional Medicine, Chinese’:ab,ti OR ‘Chinese Traditional Medicine’:ab,ti OR ‘Traditional Chinese Medicine’:ab,ti OR ‘Chinese Medicine, Traditional’:ab,ti OR ‘Traditional Tongue Diagnosis’:ab,ti OR ‘Tongue Diagnoses, Traditional’:ab,ti OR ‘Tongue Diagnosis, Traditional’:ab,ti OR ‘Traditional Tongue Diagnoses’:ab,ti OR ‘Traditional Tongue Assessment’:ab,ti OR ‘Tongue Assessment, Traditional’:ab,ti OR ‘Traditional Tongue Assessments’:ab,ti OR ‘TCM’:ab,ti OR ‘Chinese Medicine’:ab,ti) AND ('Nursing':ab,ti OR 'Nursings':ab,ti)

# Cochrane


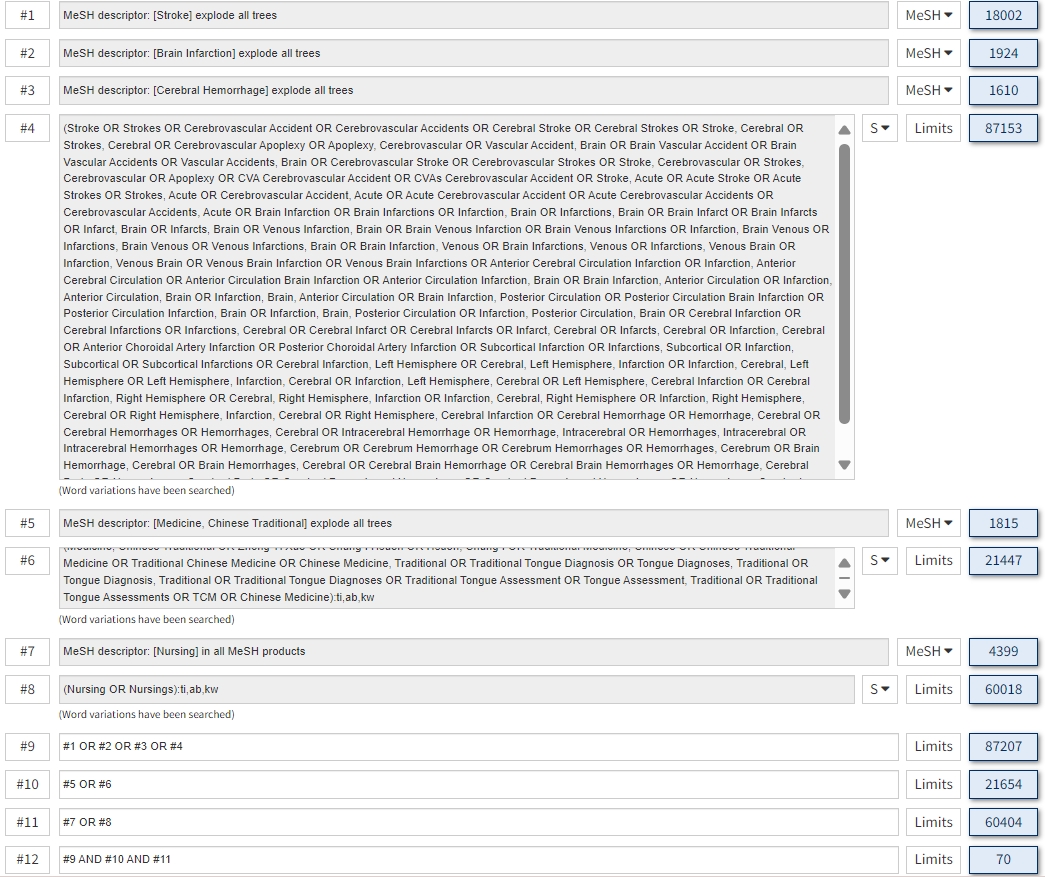


# Web of Science

TS=(Stroke OR Strokes OR Cerebrovascular Accident OR Cerebrovascular Accidents OR Cerebral Stroke OR Cerebral Strokes OR Stroke, Cerebral OR Strokes, Cerebral OR Cerebrovascular Apoplexy OR Apoplexy, Cerebrovascular OR Vascular Accident, Brain OR Brain Vascular Accident OR Brain Vascular Accidents OR Vascular Accidents, Brain OR Cerebrovascular Stroke OR Cerebrovascular Strokes OR Stroke, Cerebrovascular OR Strokes, Cerebrovascular OR Apoplexy OR CVA (Cerebrovascular Accident) OR CVAs (Cerebrovascular Accident) OR Stroke, Acute OR Acute Stroke OR Acute Strokes OR Strokes, Acute OR Cerebrovascular Accident, Acute OR Acute Cerebrovascular Accident OR Acute Cerebrovascular Accidents OR Cerebrovascular Accidents, Acute OR Brain Infarction OR Brain Infarctions OR Infarction, Brain OR Infarctions, Brain OR Brain Infarct OR Brain Infarcts OR Infarct, Brain OR Infarcts, Brain OR Venous Infarction, Brain OR Brain Venous Infarction OR Brain Venous Infarctions OR Infarction, Brain Venous OR Infarctions, Brain Venous OR Venous Infarctions, Brain OR Brain Infarction, Venous OR Brain Infarctions, Venous OR Infarctions, Venous Brain OR Infarction, Venous Brain OR Venous Brain Infarction OR Venous Brain Infarctions OR Anterior Cerebral Circulation Infarction OR Infarction, Anterior Cerebral Circulation OR Anterior Circulation Brain Infarction OR Anterior Circulation Infarction, Brain OR Brain Infarction, Anterior Circulation OR Infarction, Anterior Circulation, Brain OR Infarction, Brain, Anterior Circulation OR Brain Infarction, Posterior Circulation OR Posterior Circulation Brain Infarction OR Posterior Circulation Infarction, Brain OR Infarction, Brain, Posterior Circulation OR Infarction, Posterior Circulation, Brain OR Cerebral Infarction OR Cerebral Infarctions OR Infarctions, Cerebral OR Cerebral Infarct OR Cerebral Infarcts OR Infarct, Cerebral OR Infarcts, Cerebral OR Infarction, Cerebral OR Anterior Choroidal Artery Infarction OR Posterior Choroidal Artery Infarction OR Subcortical Infarction OR Infarctions, Subcortical OR Infarction, Subcortical OR Subcortical Infarctions OR Cerebral Infarction, Left Hemisphere OR Cerebral, Left Hemisphere, Infarction OR Infarction, Cerebral, Left Hemisphere OR Left Hemisphere, Infarction, Cerebral OR Infarction, Left Hemisphere, Cerebral OR Left Hemisphere, Cerebral Infarction OR Cerebral Infarction, Right Hemisphere OR Cerebral, Right Hemisphere, Infarction OR Infarction, Cerebral, Right Hemisphere OR Infarction, Right Hemisphere, Cerebral OR Right Hemisphere, Infarction, Cerebral OR Right Hemisphere, Cerebral Infarction OR Cerebral Hemorrhage OR Hemorrhage, Cerebral OR Cerebral Hemorrhages OR Hemorrhages, Cerebral OR Intracerebral Hemorrhage OR Hemorrhage, Intracerebral OR Hemorrhages, Intracerebral OR Intracerebral Hemorrhages OR Hemorrhage, Cerebrum OR Cerebrum Hemorrhage OR Cerebrum Hemorrhages OR Hemorrhages, Cerebrum OR Brain Hemorrhage, Cerebral OR Brain Hemorrhages, Cerebral OR Cerebral Brain Hemorrhage OR Cerebral Brain Hemorrhages OR Hemorrhage, Cerebral Brain OR Hemorrhages, Cerebral Brain OR Cerebral Parenchymal Hemorrhage OR Cerebral Parenchymal Hemorrhages OR Hemorrhage, Cerebral Parenchymal OR Hemorrhages, Cerebral Parenchymal OR Parenchymal Hemorrhage, Cerebral OR Parenchymal Hemorrhages, Cerebral) AND TS=( Medicine, Chinese Traditional OR Zhong Yi Xue OR Chung I Hsueh OR Hsueh, Chung I OR Traditional Medicine, Chinese OR Chinese Traditional Medicine OR Traditional Chinese Medicine OR Chinese Medicine, Traditional OR Traditional Tongue Diagnosis OR Tongue Diagnoses, Traditional OR Tongue Diagnosis, Traditional OR Traditional Tongue Diagnoses OR Traditional Tongue Assessment OR Tongue Assessment, Traditional OR Traditional Tongue Assessments OR TCM OR Chinese Medicine) AND TS=(Nursing OR Nursings)

# CNKI

SU%=('卒中' + '脑卒中' + '中风' + '脑血管意外' + '脑梗塞' + '脑梗死' + '缺血性卒中' + '缺血性中风' + '脑出血' + '出血性卒中' + '出血性中风') * ('中医') * ('护理' + '护理技术' + '护理模式')

# WANFANG

主题:(卒中 or 脑卒中 or 中风 or 脑血管意外 or 脑梗塞 or 脑梗死 or 缺血性卒中 or 缺血性中风 or 脑出血 or 出血性卒中 or 出血性中风) and 主题:(中医) and 主题:(护理 or 护理技术 or 护理模式)

# VIP

(M=卒中 OR脑卒中 OR 中风 OR 脑血管意外 OR 脑梗塞 OR 脑梗死 OR 缺血性卒中 OR 缺血性中风 OR 脑出血 OR 出血性卒中 OR 出血性中风) AND M=中医 AND (M=护理 OR 护理技术 OR 护理模式)
